# Independent living with Duchenne muscular dystrophy and home mechanical ventilation in areas of Japan with insufficient national welfare services

**DOI:** 10.3402/qhw.v8i0.20914

**Published:** 2013-08-26

**Authors:** Miku Yamaguchi, Machiko Suzuki

**Affiliations:** Division of Human Health Sciences, Department of Nursing, Graduate School of Medicine, Kyoto University, Kyoto Prefecture, Japan

**Keywords:** Grounded theory, health, home care, physical impairment, qualitative, social inclusion, isolation

## Abstract

In Japan, there is no national 24-hour home care system for people with severe impairments. Despite this fact, a small number of people with Duchenne muscular dystrophy on home mechanical ventilation pursue independent living. Therefore, our aim was to better understand the process by which these individuals arrived at this goal for independence (i.e., choosing to live at home in Japan instead of in special sanatoriums that provide sufficient support and care). Twenty-one participants were interviewed in 2011 and 2013. The interviews were recorded, transcribed, and analysed following a grounded theory approach. These individuals placed particular emphasis on their personal choice regarding where and how they live as well as on whom they depend. Therefore, the core element underlying participants’ goals for independent living was *self-reliant independency*. To improve their social inclusion, the strategies used by the participants to retain their autonomy in an underdeveloped Japanese welfare system by establishing relationships with people in their communities can prevent them from experiencing social isolation. This could serve as an example to their counterparts in other countries.

Duchenne muscular dystrophy (DMD) is a progressive neuromuscular disease characterized by loss of muscle strength in early childhood. “A diagnosis of DMD carries with it prolonged loss and burden; a seemingly healthy infant will grow weaker over years, lose the ability to walk as an adolescent and die as a young man. There is no known cure” (Fee & Hinton, 2011, p. 2). As patients usually experience gradual physical impairment that eventually affects their breathing, individuals with DMD become increasingly dependent in all aspects of their lives. Respiratory problems, which typically begin in adolescence, lead to respiratory failure and death by the time patients are in their 20s if respiratory assistance is not used (Lindsay & Bone, 2004). Thus, most individuals with DMD eventually require home mechanical ventilation (HMV). In medically advanced countries, treatment typically starts at the age of 16–19 years with non-invasive ventilation (Rahbek et al., 2005).

Twenty-seven special sanatoriums were established in Japan in the 1960s specifically for children with progressive muscular dystrophy, where they are admitted for medical treatment, rehabilitation services, and nursing care, as well as to attend the adjacent school. These facilities have helped to reduce the financial, physical, and emotional burden on parents (Yamada, 1999), and they have enriched the lives of persons with muscular dystrophy. Traditionally, Japanese people with muscular dystrophy continue to live in these sanatoriums until their death.

However, a worldwide independent-living movement for people with disabilities, which had its origins in the American civil rights and consumer movements of the 1960s (Zames Fleischer & Zames, 2011), gained popularity in Japan in the 1990s. People with muscular dystrophy living in sanatoriums were influenced by this movement, and they began to pursue more independent lives (Kikuchi, 2010). *Independent living* can be broadly defined as when a person, especially one with a disability, seeks to maximize his or her independence and self-determination, and lives in the community instead of in a medical facility (American Heritage Dictionary, 2012).

In a qualitative study of 19 adult patients with muscular dystrophy in Japan, participants reported that they were in the process of accepting the progression of their disease and that they were conflicted between independence and dependence (Yamaguchi, 2013). Yamaguchi (2013) reported that independent living is important for persons with physical impairments living in Japan. In Japan, if individuals with DMD live in specialized sanatoriums, they can live safe and, arguably, satisfactory lives, despite severe physical impairment. In contrast to those who advocate living at home, many individuals reported that they were able to live relatively independently in the sanatoriums due to round-the-clock support by doctors, nurses, helpers, and teachers (Ishida, 2012).

In one study, more than 90% of people with muscular dystrophy in these sanatoriums regarded themselves as “unable to live at home” (Suehara, 1999, p. 134), despite wanting to, due to insufficient in-home care. In Japan, there is no standardized system in place to provide 24-hour in-home care for people with severe muscular dystrophy. The quality of individual care often depends on the financial circumstances of individuals, as well as on local governments (Doi, 2010).

Dreyer, Steffensen, and Pederson (2010b) interviewed 19 Danish patients with DMD and found that these patients, who are provided with round-the-clock in-home care and personal assistance through Denmark's national health system (unlike in Japan), saw themselves as psychologically independent, describing themselves as practicing “independent dependency” (p. 7).

The psychological importance attached to living lives of “independent dependency” is helpful to understanding the needs, goals, and motivations of Japanese patients with DMD who are on HMV and who prefer an independent life in their communities. The aim of this study was to better understand the process by which these individuals arrived at the goal of independent living. We started with the question “Why would these individuals prefer to live in a community that provides relatively poor social welfare services over the specially designed and high-quality sanatoriums available in Japan?” As DMD is a progressive disease, we decided to focus on concepts related to changes in participants’ notions of independence according to the disease's progression. We hope to extend our understanding of the concept of independence of Japanese patients with DMD on HMV through dehospitalization.

Some theories already pertain to independence in patients with progressive muscular dystrophy (Dreyer, Steffensen, & Pederson, 2010a, 2010b; Ishida, 2012; Yamaguchi, 2013). Although these participants differed in their disease type and living environments, all of them reported living independently. Therefore, according to the samples and findings of these previous studies, we decided to focus our inquiry on individuals for whom living independently was the biggest challenge—adults with DMD on HMV (invasive or non-invasive, intermittent, or continuous)—who chose to live at home instead of in sanatoriums in Japan.

The data analysed in this article were taken from a larger study that explored the concept of independence in Japanese patients with progressive muscular dystrophy and the experiences of their parents of bringing them into adulthood. We modified the inclusion criteria to sample only patients with DMD not living in sanatoriums at the time of interview. We also interviewed additional participants to collect new data.

## Method

Grounded theory seeks to understand human behavior in a social context (Glaser & Strauss, 1967) and is an appropriate approach for discovering rather than verifying theory within textual data. This approach is used to study phenomena without a well-formulated prior theory; an inductive process analyses data as they are being collected, as opposed to conducting all analyses after data collection is complete. This generates *substantive theory*, which is “a strategic link in the formulation and generation of grounded formal theory” (Glaser & Strauss, 1967. p. 79), which is designed for a specific area of concern, as opposed to the formal theory, which is applied more broadly and abstractly. The modified grounded theory approach (M-GTA; Kinoshita, 1999, 2003) focuses on organizing substantive theory for practical utilization. Therefore, this method is more suited to our purpose of conceptualizing a process from a substantive perspective.

Furthermore, M-GTA is different from the classic grounded theory in the strict coding procedure. In this study, the coding procedure was guided by the M-GTA, which provides explicit instructions in coding (for recent examples of this approach, see Asakura & Watanabe, 2011).

### Theoretical sampling procedure

Grounded theory usually calls for theoretical sampling, as opposed to probabilistic sampling, in order to best compare new findings with the previous findings (Glaser & Strauss, 1967; Kinoshita, 1999, 2003). We thus selected two comparison groups to allow for concurrent data collection and analysis. We first recruited participants from a group of outpatients from a muscular dystrophy sanatorium that specializes in HMV. As interregional discrepancies in the welfare system may affect individuals’ ability to live independently, we selected participants from as wide a geographical area as possible. We then contacted 122 independent-living centers that provide support for individuals with impairment and that are sponsored by the Japanese government, and asked them to refer clients with DMD who use HMV.

#### Data collection

All interviews were conducted by the first author. The interviewer introduced herself and explained that she was interested in studying the independent life of people with DMD who use HMV and who live at home. Semistructured interviews (*N*=21) were conducted in Japanese by the first author in the participants’ homes. The opening question was “Please tell me your background with DMD.” The participants were asked to speak freely about their past experiences. They were then asked about their present independent living. If needed, participants were prompted with questions such as “Please tell me why you started living independently at home” and “What has been your experience with independent living?” To clarify answers and elicit in-depth descriptions, the interviewer sometimes asked, “Can you tell me more about that?” Data were collected in January 2011 and April 2013. Although the interview was conducted only once per participant, some interviews were paused and postponed two or three times due to participants’ physical conditions.

The interviews were audio recorded, translated from Japanese into English, and then back-translated by bilingual translators. The back-translated interview data were checked by an English native speaker, who confirmed the appropriateness of the translation in conveying participants’ original information.

#### Ethical considerations

This study was approved by the Medical Ethics Committee of Kyoto University (Approval No. 920). Our study conformed to the principles set by the Declaration of Helsinki. Informed consent was obtained both verbally and in writing. All participants were informed that their participation was voluntary and that they could withdraw from the study at any time without penalty. The participants’ physical condition was considered the first priority during the interview. The interview was suspended and rescheduled if the participants required it.

#### Analysis

The grounded theory approach suggests using constant comparison to analyse qualitative data (Glaser & Strauss, 1967; Kinoshita, 1999, 2003). This approach is useful in that data are analysed as they are being collected; after each interview was transcribed, the data were coded, generating theory inductively. Data were coded by their content with the purpose of revealing substantive theory that emerged, as well as for the purpose of achieving a constant comparison of incidents and concepts that emerged from subsequent interviews (Glaser & Strauss, 1967; Kinoshita, 1999). Codes were continuously reread for in-depth analysis of data. Codes with similar meanings were grouped to allow for the development of concepts. In accordance with the M-GTA, we used an “analyzing worksheet” to categorize the codes according to the concepts. The analyzing worksheets also included ideas for concept names and memos for interpretation from codes.

A recognized qualitative data management program, NVIVO8 (QSR International), was also used to output all codes to the worksheets. This program was also used to manage all data and to confirm that there was no overlapping of data in the categories. The relationships between categories were organized and theorized. The structure of the relationship between categories was clarified, identifying the main categories. As we continued, theoretical saturation became more complete. A more abstract elevated analysis was conducted through which a core category, named *self-reliant independenc*y, emerged. Finally, we conducted one new interview to confirm theoretical saturation. We then conducted three feedback interviews with participants to validate the theory and determine whether these concepts were adequate for interpretation.

## Results

Table I shows the characteristics of participants. Twenty-four interviews were conducted overall. All of the participants were men (DMD disproportionately affects men) who had previously lived at home during childhood, were subsequently admitted into the muscular dystrophy sanatorium, and finally moved back home at the time of the study. Participants ranged in age from 22 to 57 years, and they received an average of 12.8 h of help per day. Most of them received some support from family members or unpaid volunteers. Despite the difficulties they encountered in living independently, all of them were content with their present state.

**Table I:** Participant characteristics (*N=*21).

Male/female	21/0
Age in years (mean±SD)	31.4±10.8
Religious beliefs	
No	19
Yes	2
Occupation	
Staff at independent-living center	12
Activist	4
None	5
Helper services received (hours per month)	
Mean±SD	385±159.1
Range	190–600
Interview length (min)	
Mean±SD	68.2±48.0
Range	42–138
Helped by unpaid volunteers	
Yes	15
No	6
Helped by family members		
Yes	19
No	2
Age at which they left the sanatorium	
Mean±SD	26.5±8.3
Range	20–49

**Figure 1:**
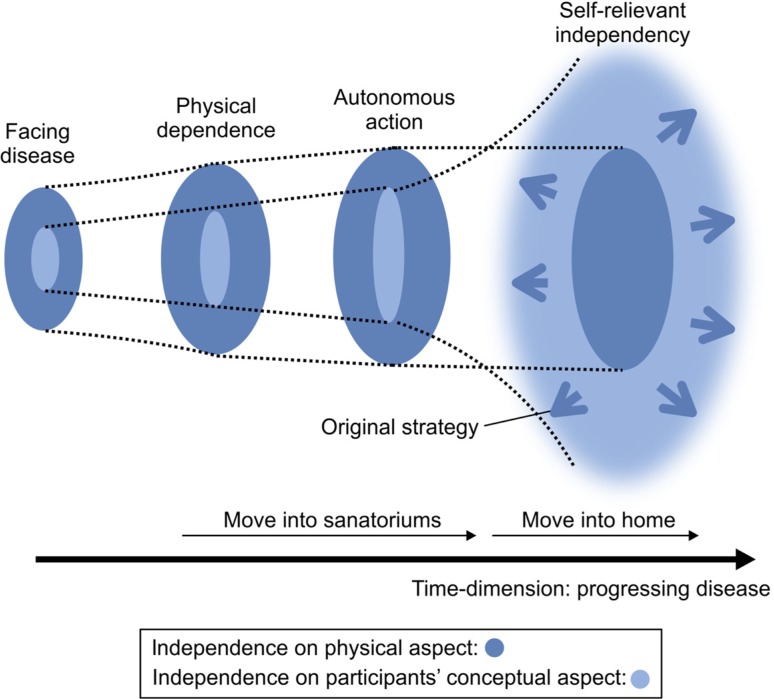
Self-reliant independency. The concepts converging on the core category as the study participants’ data unfolded are shown. Their expansion of the concept of independence changed with disease progression. Beginning with *facing disease*, followed by *physical dependence* and then *autonomous action*, the study participants discovered their ability to live independently within their physical limits. After that, the concept of independence was condensed as self-reliant independency, which meant having more autonomy in decision making than in the sanatoriums. In the case of participants, this was exemplified by moving out of the sanatoriums. Participants obtained independence without being limited by physical dependency. *Original strategy* then helped them to maintain their independent living and broaden the possibilities for their self-reliant independence.

The interview data yielded 142 codes that fell into five categories, with *self-reliant independency* identified as the core category (see Figure 1). Four categories (*facing disease*, *physical dependence*, *autonomous activity*, and *original strategy*) were found that are related to self-reliant independency. The following section will discuss each of these categories, beginning with the core category, self-reliant independency.

### Self-reliant independency

The core category of self-reliant independency emerged as the final goal of independent living. Participants with severe impairments have experienced disease progression since childhood. Due to increasing physical dependency, participants seek more autonomy in their daily lives in terms of making decisions, which seems to be the main reason for them choosing to live at home. What makes the core category of self-reliant independency so poignant was that even though participants recognized the progression of their disease and the necessity of depending on others physically, they chose to pursue self-reliant independence.

Certainly, life in the sanatoriums is easy and safe. But … anyone would want to live more freely. Don't you think? Of course, we with DMD can't keep on living without relying on others. But … but I want to decide by myself about my life. I decide and judge by myself as best I can. I think maybe that is to be an adult. And taking responsibility for my matters, that is my independent living. (Participant 21)

If you need to be free from only physical disability, you would be satisfied with life in the sanatoriums. We can also play powerchair football in there. Besides, parents need not worry about their sons. But … have I got to live in there throughout my life? (Participant 3)

Once I moved out of the hospital, I experienced enrichment of my life at home. Of course, my appearance has changed from childhood. I have used the respirator from my teenage years; now the tracheostomy tube protrudes from my neck. Besides, I know that life at home will increase medical risk more than hospitalization (in a sanatorium). But now I can't imagine going back to the sanatorium. (Participant 3)

In order to assert their self-reliant independency, they have tried dehospitalization and returned home despite complete or almost-complete physical dependence and the underdeveloped Japanese welfare system. It seems that participants found their lives enriched through living independently in their own way. Indeed, as one participant said,

Now I can live with hope … independence … real independence. That's all. (Participant 11)

### Facing disease

This category is the root of the other three categories. It refers to participants’ acknowledgment and, consequently, acceptance of what having DMD entails. Facing refers to acknowledging the progression of their disease and what will happen to their bodies. As they became older, participants began to face their disease. While other children grew into healthy youths, participants experienced gradual physical function decline. Accepting that they would be dependent on HMV to breathe was a major event in their adolescence. Their acceptance of the disease progression occurred by facing and understanding the disease. As one participant stated,

I have gradually come to understand my disease. Here is my disease, now I know. I have experienced becoming less and less able. (Participant 8)

Another participant said,

Until I started to use the respirator, it had been really hard to breathe. Finally, I reached the limit of breathing on my own and began to use a ventilator. At first, I struggled to use it, I couldn't judge whether it fitted or not. However, my breathing became easy after I adapted myself to it. Using a ventilator is part of understanding our disease. (Participant 9)

The participants have gradually accepted living with physical impairment, which was a result of facing their disease.

### Physical dependence

While growing up, participants were made aware that they would gradually require more physical assistance. As a result, participants’ relationships with their peers in school changed from reciprocal friendships to supportive relationships in which they receive more help. They have accepted that depending on others was their only way to live with DMD. This physical dependence clashed with their desire to maintain their reciprocal friendships in school. It is possible that this conflict, coupled with their increasing physical dependence, led some participants to opt for admission into sanatoriums. As the participants stated,

I was satisfied with my school life. For example, my friends helped me to use the toilet. They supported me in sitting on a chair and using a chamber pot. In that way, I received a little help from others. During my childhood, I was so happy to get support. (Participant 16)

When I was a little boy, I was able to continue schooling due to being helped by teachers and other children. But while I grew up and my impairment increased in severity, I began to be not as good at asking others for help. It was increasingly difficult for me. Then I decided to go into the muscular dystrophy sanatorium of my own free will. (Participant 10)

### Autonomous action

In the sanatoriums, participants perceived that they could obtain their bodily autonomy despite being entirely dependent on caregivers. Autonomous action precipitated the verification of them being able to manage their daily activities and some matters surrounding them. In this situation, participants obtained physical operability for their daily activities, which led to their accepting their future life of severe impairment. In the sanatoriums, they were granted autonomous action by being provided with a safe and comfortable environment with round-the-clock special care and support. Participants also learned to establish relationships with special care staff and helpers through the process of experiencing autonomous action, which is a meaningful step toward their own independence. Indeed, according to one participant,

In the sanatorium, there were special helpers. It was completely different. I could do everything without physical burden. My life there was much easier than it used to be, on the physical side. (Participant 11)

Another participant stated,

In the sanatorium, I learned that I can manage my life, even if I was in a wheelchair or used a ventilator. I can live with my physical impairment … being hospitalized in youth can be regarded as a meaningful step toward learning how to go on relying on others. Children with the same disability, older and younger, were gathered and lived together … I have learned in there how to receive support from others following the example of senior patients. We learned to get hold of help from caregivers such that we add as little burden to them as possible … selected different persons depending on its purpose. We used properly procured help depending on the situation. These experiences were helpful for me to live with being supported by various people in the community. (Participant 9)

Although participants experienced autonomous living in the sanatoriums, they wanted even more independence, which consisted of having more autonomy in decision making than in the sanatoriums. They assert that independence is obtained through acting by their own will and choice in everything (i.e., self-reliant independency). It was also important that they discovered their definition of independent living in community life, which is built on supportive relationships with caregivers. It is noteworthy that it was in the hospital that they learned to build helpful relationships with others on whom they physically depended. They used this experience of autonomous action with other-reliant independence in hospital life to prepare for community life.

### Original strategy

The participants’ experiences with dealing with their disability have helped them to acquire the strategies needed to maintain life in a community in Japan. This category refers to the strategies they used to maintain and simultaneously extend their autonomy. As the participants stated,

(I have learned) Not to exert myself about everything. If I catch a cold, I have to call my doctor immediately and take a medicine already prepared beforehand. (I have) To keep stock of household medicines. (I cannot) Overlook any little change in my body. (I had to) Establish my own empirical rules to deal with matters by myself as much as possible and ask for assistance from my helpers. Or I will usually consult my home-visit-nurse or physical therapist or doctor. (Participant 21)

I was the first to be involved in the launch of a volunteer organization. Up until then, there hadn't been any students with assistance needs at the university. A friend with pretty severe cerebral palsy in the same school year and I tried to organize a volunteer group. (Participant 15)

These strategies brought them confidence in being able to maintain independent living and broadened the possibilities for their self-reliant independency; they have established their own ways of physical management and obtained peer support in some cases. Some of them also reported maintaining good relationships with their parents. Due to these strategies, they have been able to shoulder more responsibilities of self-reliant independent living, which led to the consolidation of self-reliant independency.

## Discussion

In Japan, individuals with DMD can choose to live in their homes or in muscular dystrophy sanatoriums. All study participants initially chose to live in sanatoriums when the progression of their disease first became too onerous for friends and family to manage. While living in the sanatoriums, they learned how they could be autonomous while still being dependent on caregivers. Following this realization, they all left the sanatoriums in order to achieve self-reliant independency.

Dreyer et al. (2010b) stated that the physical dependency of patients with DMD on HMV is not inconsistent with independence; rather, it is an interdependence.^1^ Ishida (2012) found that patients with muscular dystrophy living in sanatoriums report perceiving themselves as living independently despite living a life based on physical dependence on caregivers. In this study, we also saw how participants reconciled physical dependence with autonomy of action. While their physical actions were dependent on others, psychologically, they did not feel dependent; instead, they achieved an “active life despite physical impairment” (Dreyer et al., 2010b, p. 7).

Although our findings were similar to those of Dreyer et al. (2010b) and Ishida (2012) with regard to participants’ views on independence with physical dependency, a major difference was that the participants in the other two studies maintained this independence within helpful surroundings for physical dependency, while our participants acted autonomously in non-institutionalized surroundings. Therefore, the participants in this study evidently viewed independence differently, choosing to work with a comparably lower level of care and support in order to materialize their original self-reliant independency.

We label the independent living sought by participants in this study as *self-reliant independency*, as opposed to the independent living reported in previous studies, because the latter seems to refer to the refusal of any support, whereas the former acknowledges physical dependence. This is the first time that this distinction has been found in a sample of Japanese individuals with DMD.

The finding of the desire for self-reliant independency in Japanese patients with DMD is particularly notable because Japan is a collectivistic, interdependent society (Maeda, Hagihara, Kobori, & Nakayama, 2006). Individuals are socialized to adjust to the relationships to which they belong, occupying and fulfilling their assigned roles, and engaging in appropriate actions (Voltz, Akabayashi, Reese, Ohi, & Sass, 1998). In this study, participants first saw their roles as patients with physical impairments, making it therefore appropriate for them to accept care from special caregivers in sanatoriums. However, the participants in this study all came to reject this role, deciding that independence, as they saw it, could not be obtained while living in sanatoriums. They consequently broke free of this role by discharging themselves from the sanatoriums and moved back into the community.

If adults living with progressive muscular dystrophy find that they cannot fulfill their vision of having independent lives within the sanatorium system, then there is an urgent need for Japan to establish the 24-hour in-home care systems available in countries such as Denmark. However, before implementing such a system, it should be noted that these systems are not without disadvantages, the most serious of which is social isolation. While adults with DMD are living longer lives due to medical and technological advances, patients’ social isolation can “last for over a decade” (Gibson, Zitzelsberger, & McKeever, 2009, p. 565). Bothwell et al. (2002) reported that social isolation increases for many adults with DMD as they age, and Lindsay and McPherson (2011) advocate enhancing the social inclusion of persons with disabilities to counteract isolation. In this study, participants have established supportive relationships with others in the community as a strategy to combat physical immobilization in the insufficient Japanese welfare system. This might alleviate their social isolation from the community. Additionally, as shown in Table I, most of our participants worked at independent-living centers as consultants for persons with severe impairments, further helping them to live independently. These patients’ adaptations to achieve the goal of living independently in Japan could provide their counterparts in other countries with helpful and relevant knowledge on how to prevent social isolation while being severely impaired physically.

## Study limitations

Our goal was to better understand the process by which these individuals arrived at the goal of independent living in a population with severe physical impairment—adults with DMD who use HMV and live in their homes or in the community in Japan. Our findings provide insight into the support and difficulties experienced by these participants and highlight the insufficiency of community-based services for those who reject life in a sanatorium. However, these findings may not be generalizable to all persons with DMD who use HMV in different countries with different cultural backgrounds or with different healthcare systems. Another potential limitation is that all our participants were male, while the interviewer was female. Participants might be less inclined to discuss emotional or sexual issues with an interviewer of the opposite sex.

## Conclusion

Our study shows that *self-reliant independency* is unique to Japanese people with DMD on HMV. It is obtained through the participants’ experience with disease progression, physical dependence, and autonomy. This concept contrasts with the ascribed independence that others with DMD have found satisfying. The present participants were satisfied with establishing their own independence by adapting to the underdeveloped welfare service in Japan. The participants’ ability to lead independent lives and have good relationships with others and regular occupations as consultants can prevent social isolation and serve as an example to other DMD patients living outside institutions.

## Footnotes

1 The meaning of *interdependence* was not clarified in the study by Dreyer et al. (2010b). We have assumed the meaning of this word to be “care receiver and giver being dependent on each other,” that is, the caregiver is dependent on the receiver for decision making while the receiver is dependent on the caregiver for physical aspects.

## References

[CIT0001] American Heritage Dictionary (2012). The American heritage dictionary.

[CIT0002] Asakura K, Watanabe I (2011). Survival strategies of male nurses in rural areas of Japan. Japan Journal of Nursing Science.

[CIT0003] Bothwell J. E, Dooley J. M, Gordon K. E, MacAuley A, Camfield P. R, MacSween J (2002). Duchenne muscular dystrophy–Parental perceptions. Clinical Pediatrics.

[CIT0004] Doi K (2010). Kaigo Jikan wo Fuyasu Koushou ga Nanka. Nanbyou to Zaitaku Care.

[CIT0005] Dreyer P. S, Steffensen B. F, Pederson B. D (2010a). Life with home mechanical ventilation for young men with Duchenne muscular dystrophy. Journal of Advanced Nursing.

[CIT0006] Dreyer P. S, Steffensen B. F, Pederson B. D (2010b). Living with severe physical impairment: Duchenne's muscular dystrophy and home mechanical ventilation. International Journal of Qualitative Studies on Health and Well-Being.

[CIT0007] Fee R. J, Hinton V. J (2011). Resilience in children diagnosed with a chronic neuromuscular disorder. Journal of Developmental and Behavioral Pediatrics.

[CIT0008] Gibson B. E, Zitzelsberger H, McKeever P (2009). “Futureless persons”: Shifting life expectancies and the vicissitudes of progressive illness. Sociology of Health & Illness.

[CIT0009] Glaser B. G (1978). Theoretical sensitivity: Advances in the methodology of grounded theory.

[CIT0010] Glaser B. G, Strauss A. L (1967). The discovery of grounded theory: Strategies for qualitative research.

[CIT0011] Ishida M (2012). Kin-dystorophy Byoutou ni Kurasu Seijin-Knjya-tati no Seikatu Keiken—Jikan ni Kakawaru Keiken no Genshougakuteki Kijyutu. Nihon Nanbyo Kango Gakkaishi.

[CIT0012] Kikuchi M (2010). Kin-dystrophy Byoutou no Hensen: Kin-dystrophy Byoutou deno Ryouyou wo Meguru Kenkyuu no Houkousei wo Saguru. Tokyo Jikeikai Medical Journal.

[CIT0013] Kinoshita Y (1999). M-GTA—Modified grounded theory approach—Grounded theory approach: Shituteki jissho kenkyu no saisei.

[CIT0014] Kinoshita Y (2003). M-GTA—Modified grounded theory approach—Grounded theory approach: Shituteki kenkyu he no Sasoi.

[CIT0015] Lindsay K. W, Bone I (2004). Neurology and neurosurgery.

[CIT0016] Lindsay S, McPherson A. C (2011). Strategies for improving disability awareness and social inclusion of children and young people with cerebral palsy. Child Care. Health and Development.

[CIT0017] Maeda Y, Hagihara A, Kobori E, Nakayama T (2006). Psychological process from hospitalization to death among uninformed terminal liver cancer patients in Japan. BMC Palliative Care.

[CIT0019] Rahbek J, Werge B, Madsen A, Marquant J, Steffensen B. F, Jeppesen J (2005). Adult life with Duchenne muscular dystrophy: Observations among an emerging and unforeseen patient population. Pediatric Rehabilitation.

[CIT0020] Suehara M (1999). Dare no Nan no Tame no Nyuin Care wo Mezasu Bekika. Kouiseisho Seisin Shinkei Sikkan kenkyu Kin-dystorophy Kanjya no QOL no Koujyou ni Kansuru Houkokusho.

[CIT0021] Voltz R, Akabayashi A, Reese C, Ohi G, Sass H. M (1998). End-of-life decisions and advance directives in palliative care: A cross-cultural survey of patients and health-care professionals. Journal of Pain Symptom Management.

[CIT0022] Yamada T (1999). Zenshin Ugokazu [Immobilization of the whole body].

[CIT0023] Yamaguchi M (2013). Tiiki de Seikatu suru Sinkousei-Kin-dystorophy-kanjya ga Jiritu ni mukau Purosess no Kentou. Nihon Kango Kagaku Gakkaisi.

[CIT0024] Zames Fleischer D, Zames F (2011). The disability rights movement: From charity to confrontation (updated ed.).

